# Interspecific and Intraspecific Artificial Insemination in Domestic Equids

**DOI:** 10.3390/ani13040582

**Published:** 2023-02-07

**Authors:** Diana Fanelli, Rebecca Moroni, Carlotta Bocci, Francesco Camillo, Alessandra Rota, Duccio Panzani

**Affiliations:** Veterinary Sciences Department, Pisa University, Via Livornese, San Piero a Grado, 56124 Pisa, Italy

**Keywords:** hybrids, pregnancy rates, mule, hinny, artificial insemination

## Abstract

**Simple Summary:**

Among equids, the mule (jackass stallion × mare) is the most common hybrid, followed by the hinny (horse stallion × jenny). This study describes the outcome of inseminating mares and jennies with either fresh horse or donkey semen of proven fertility. Pregnancy rates in horse females were significantly higher than in donkey females, while horse and donkey males did not affect pregnancy rates. Overall, intraspecific pregnancy rates were significantly higher than interspecific ones.

**Abstract:**

Horses and donkeys differ phenotypically and karyotypically, although they can interbreed freely. Eight Standardbred mares and nine Amiata donkey jennies were included in the study. Semen was collected from two horses and two donkey stallions of proven fertility. A first pregnancy diagnosis was performed on day 10 after ovulation and repeated every day until embryo detection or until day 16. Irrespectively of the sire species, pregnancy rates in horse females (20/30, 66.7%) were significantly higher than in donkey females (19/70, 27.1%) (*p* < 0.05), while horse and donkey males did not affect pregnancy rates. Comparing overall intraspecific and interspecific AI, pregnancy rates were 25/37 (67.6%) and 14/63 (22.2%), respectively (*p* = 0.0001). The lowest pregnancy rate was obtained when inseminating jennies with horse stallion semen (8/49, 16.3%). No statistical differences were found when comparing embryo diameters, day at first pregnancy diagnosis, or in vitro embryo morphological quality among groups. In this study, much poorer results were obtained with jennies than with mares. Interspecific AI resulted in lower pregnancy rates than intraspecific Al, and AI to produce hinny hybrids resulted in the lowest pregnancy rate. Further studies are required to better understand the mechanism involved in such different outcomes in relation to intra- and interspecific breeding in domestic equids.

## 1. Introduction

In reproductive biology, a hybrid is an offspring produced from a cross between parents of different species or sub-species and is usually sterile. Hybridization between different mammalian species has been reported to occasionally occur in nature (e.g., sheep × goat, tiger × lion, dog × wolf, etc.); however, apart from the mule, it is far more commonly the result of intentional breeding programs [1].

The mule (jackass × mare, 2n = 63) and the hinny (horse stallion × jenny, 2n = 63) are the most common equine hybrids since their parents (*E. Caballus*, 2n = 64 and *E. Asinus*, 2n = 62) are the only equine species that have been truly domesticated. For centuries, mules have been bred for use in agriculture, the military, or for leisure activities, thanks to the mixture of physical and mental attributes of the two parental species. Mules have played an important role in military actions, for example, they were used in World Wars I and II to carry artillery, food supplies, and even wounded soldiers on the battlefield [2,3,4].

Due to mechanization, mules have lost their traditional role and are now used both for hippotherapy and leisure riding. However, the mule is still irreplaceable for deforestation work in inaccessible mountain areas and the management of big bovine herds, above all in South America [5]. In contrast, hinnies seem to be much less common and diffused [6,7].

There are many different equine hybrid combinations, such as breeding between Przewalski (*Equus Prezwalskii*, 2n = 66) and domestic horses (*E. Caballus*, 2n = 64) horses, wild (e.g., *E. Hemionus*; 2n = 54) and domestic (*E. Asinus*; 2n = 62) asses, as well as between various subspecies of zebras (*E. Greuyii*, 2n = 46; *E. Burchelli*, 2n = 44; *E. Zebra*, 2n = 32) [6]. When breeding has occurred between domestic horses and wild asses or zebra species, pregnancies and foals were obtained only when the female of the partnership was the horse [8]. However, conception does not occur in attempting reciprocal crosses by mating a male domestic horse with a wild female donkey or a zebra [8] and significantly lower conception rates were achieved when attempting to produce hinnies instead of mules [6].

This disparity in pregnancy rates between reciprocal hybrid mating in equids is also seen in other species. For example, high fertilization rates have been reported when inseminating female goats with ram semen; however, much lower fertilization rates occurred when inseminating ewes with goat semen [9,10]. Similarly, inseminating rabbits with hare sperm led to significantly higher pregnancy rates than reciprocal crosses [11]. The mechanisms responsible for these large differences remain unknown.

Over the years, the use of artificial insemination (AI) has enabled a single male to mate with many females, even at large distances, thus reducing the risk of transmission of venereal diseases and trauma [12]. Whilst the use of intraspecific (within the same species) AI in horses and donkeys has been extensively studied [13,14,15,16,17], interspecific (within different species) AI has been much less investigated.

Intraspecific insemination with fresh semen in equine species leads to high pregnancy rates [13,14,15,18]. However, the results are significantly lower in asinine species, even when using fresh semen collected from jacks of proven fertility [16,19,20,21].

A few studies on a limited number of animals have reported high pregnancy rates of 71.4–93% when inseminating mares with fresh donkey semen of proven fertility [16,22,23]. The only paper on interspecific artificial insemination of horse stallion × jenny reported a very low pregnancy rate of 14% [24].

To the best of our knowledge, no studies have directly compared the fertility of intraspecific and interspecific crosses using the same males and females in all different combinations. Therefore, this study aimed to describe the outcomes of a controlled experiment exploring the artificial insemination of mares and jennies with either pooled horse or donkey fresh-extended semen. The hypothesis was that fertility, expressed as the presence of embryo vesicles between 10 and 16 days after ovulation, was higher in intraspecific than in interspecific mating.

## 2. Materials and Methods

This study was carried out at the Department of Veterinary Sciences, Pisa University (43°41′00″ North, 10°21′00″ East) and was approved by the Ethical Committee of Pisa University (approval 41/2022) after informed consent was obtained from the animal supervisor.

### 2.1. Donor Mares

Eight Standardbred mares aged from 9 to 21 years (mean ± SD, 16 ± 4.93 years) and nine Amiata donkey jennies aged from 7 to 15 years (mean ± SD, 12.36 ± 2.5 years) were included in the study. All animals were healthy, with body condition scores between 3–4 out of 5 [25], cyclic, and had a history of normal fertility. Mares and jennies were kept in paddocks. Mares were fed mixed-grass hay and water ad libitum, and the diet was supplemented once a day with commercial horse feed (moisture content 12.2%, protein 16.3%, oils and lipids 1.7%, cellulose 6.8%, ash 2.7%, sodium 75 mg/kg; Equifioc, Molitoria Val di Serchio). Jennies were fed mixed-grass hay twice a day and water ad libitum. Quality details of the hay cannot be provided, but none of the animals were affected by contextual pathologies such as stomatitis, dysphagia, or FFWS linked to the poor quality of the hay, suggesting the adequacy of the provided hay [26]. Animals are checked weekly and body condition scores of both mares and jennies were monitored once a month and did not change during the study.

### 2.2. Semen Collection and Processing

Two Monterufolino ponies and two Amiata donkey stallions, which were healthy, fertile, and had body condition scores of three [25], were employed. Stallions of both species were stabled in boxes and fed mixed-grass and alfalfa hay twice a day and water ad libitum; the horse stallions received a daily supplement of 1 kg of oats. Body condition scores of both horse and donkey stallions were monitored once a month and did not change during the study.

The stallions were submitted to semen collection using a Colorado-model artificial vagina (ARS, Chino, CA, USA) and jumping on a phantom (horse stallions) or estrous jenny (donkey stallions). After collection, the semen was filtered with sterile gauze to remove the gel fraction, then the volume was calculated, and the sperm concentration was evaluated using a spectrophotometer (Accucell, IMV Technologies, L’Aigle, France). Sperm subjective motility was estimated immediately after dilution 1:1 (*v*/*v*) in INRA96 (IMV Technologies, France) that was pre-warmed at 37 °C. Each insemination dose consisted of 1 × 10^9^ total spermatozoa at a 1:1 ratio for the two stallions of the same species, which was diluted with INRA96 to obtain a final volume of 10 mL and with subjective total motility > 85%.

### 2.3. Ovarian Activity Monitoring, Artificial Insemination, and Pregnancy Diagnosis

Transrectal ultrasound (US) (Esaote MyLab^®^ DeltaVET equipped with a 7.5 Mhz probe, model LV153) was performed daily between the beginning of estrus and detection of ovulation. Upon detection of a 35 mm follicle in the mares and 30 mm follicle in the jennies, estrus females were assigned to a crossover design study for intraspecific or interspecific AI. AI was performed every 48 h until ovulation occurred. When a post-AI uterine reaction was detected the day after AI, animals were treated with lactate ringer uterine flushing and oxytocin (20 UI, Neurofisin, FATRO SPA, Ozzano dell’Emilia, Italy). US was carried out for pregnancy diagnosis starting 10 days after ovulation and daily until the embryonic vesicle was detected or until day 16 after ovulation. Animals were treated with 3 mg/im of the prostaglandin F2alpha analog alfaprostol (Gabbrostim^®^, Vetem Spa, Monza-Brianza, Italy) after uterine flushing for embryo recovery or at 16 days after ovulation when not pregnant. A total of 100 cycles were included in the study: 30 in mares and 70 in jennies, respectively. Horse and donkey semen was employed in 16/30 and 14/30 cycles of mares, respectively, and in 49/70 and 21/70 cycles of jennies, respectively.

### 2.4. Embryo Recovery and Evaluation

**Figure 1:**
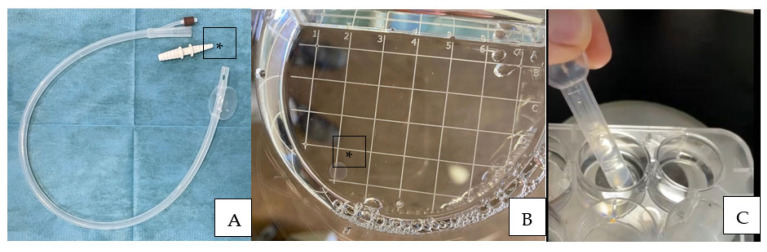
One-way 36 Fr silicon cuffed catheter used for uterine flushing for embryo recovery (**A**). Note that the plastic fitting [*] was removed after RL irrigation to recover the fluid without damaging the embryo. (**B**) EZ-Way Filter without the lid, [*] note one of the recovered embryos. (**C**) Modified 3 mL plastic Pasteur pipette for embryo manipulation.

At the first positive pregnancy diagnosis, uterine flushing for embryo recovery was performed using a modified technique with a one-way 36 Fr silicon cuffed catheter (Minitübe, Tiefenbach, Germany) and an EZ-Way Filter with the lid removed (Minitübe, Tiefenbach, Germany) (Figure 1A,B).

The uteruses were flushed with either one or two liters (for jennies and mare, respectively) of Ringer lactate that was pre-warmed at 37 °C (Galenica Senese, Siena, Italy) [27]. After flushing, both mares and jennies were treated with PGF2 alpha to induce luteolysis and a new estrus cycle, as previously specified.

**Figure 2:**
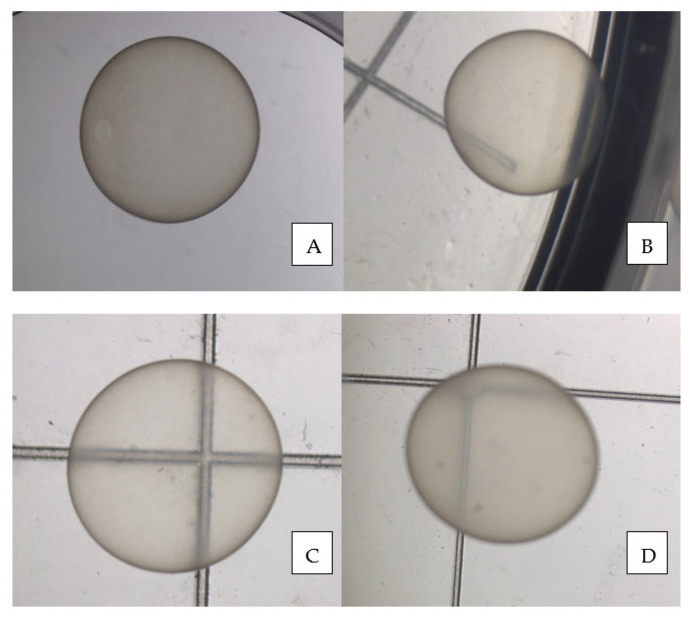
Grade 1 horse (**A**), donkey (**B**), mule (**C**), and hinny (**D**) day 11–12 embryos.

The recovered embryos were submitted to morphological evaluation [28] under a stereomicroscope (Figure 2), washed four times in ringer lactate using a modified and cut 3 mL plastic Pasteur pipette (BRAND^®^ pipette, Merck KGaA, Darmstadt, Germany) (Figure 1C), frozen, and stored for further analysis.

### 2.5. Statistical Analysis

IBM^®^ SPSS^®^ Version 26 for MacOsX was used for all the statistical analyses. The Shaphiro-Wilk normality test was used to determine the normality of distribution of the interspecific and intraspecific ultrasound embryo diameters for the day of pregnancy diagnosis, species of mother, species of father, and embryo species.

An ANOVA GLM test was performed to highlight statistical differences among embryos of different combinations of father and mother species (horse × horse, donkey × donkey, horse stallion × jenny, donkey stallion × mare).

To identify variables associated with pregnancy rates, univariable analysis of the data was performed using Fisher’s exact test with Bonferroni correction to compare cycle, presence of uterine fluid, and pregnancy rates between hybrid and non-hybrid embryos, among mother (mare and jenny) and father (stallion or jack) species, insemination combinations, and embryo species (stallion-mare, jack-jenny, stallion-jenny, jack-mare). Data were considered significantly different with *p* < 0.05. For data showing significant differences from the univariable analysis, a forward stepwise multivariable logistic regression model based on the Wald statistics criterion of a *p*-value smaller than 0.10 was used to establish significant predictors of pregnancy rates.

## 3. Results

Overall, 39/100 (39%) pregnancies were recorded during the study, of which 5/39 (12.8%) were twin pregnancies and 34/39 (87.2%) were singletons. Twin pregnancies were recorded in mule (n = 1), horse (n = 2), and donkey (n = 2) genotypes, respectively.

One pregnancy was detected at only day 16 after ovulation when the embryo vesicle was 20 mm. In this case, the embryo was excluded from diameter evaluation at first detection and was not recovered via uterine flushing. One of the embryos was broken during the recovery procedure and was excluded from the morphology evaluation.

The presence of uterine fluid one day after breeding was similar between the different combinations: 2/16 (12.5%) horse × horse, 3/49 (6.1%) horse stallion × jenny, 1/21 (4.8%) donkey × donkey, and 3/14 (2.1%) donkey stallion × mare, (*p* > 0.05). The occurrence of post-breeding endometritis was not different between intraspecific (3/37, 8.1%) or interspecific AI (6/63, 9.5%) (*p* > 0.05) and did not differ between mares (5/30, 16.67%) and jennies (4/70, 5.7%) (*p* > 0.05). No effect of the repeated insemination and flushing procedure was observed in mares nor in jennies (*p* > 0.05).

### 3.1. Pregnancy Rates

Irrespectively of the sire species, the pregnancy rate in female horses (20/30, 66.7%) was significantly higher than in female donkeys (19/70, 27.1%) (*p* < 0.05). On the other hand, the use of horse or donkey semen did not affect the pregnancy rate: 22/65 (33.8%) and 17/35 (48.6%), respectively. Table 1 shows the pregnancy rates according to the genotypes of the conceptus.

Comparing overall intraspecific versus interspecific AI, the pregnancy rates were 25/37 (67.6%) and 14/63 (22.2%), respectively (*p* = 0.0001).

The logistic regression model was statistically significant: χ^2^(2) = 29.893, *p* < 0.0005. The model explained 35.0% (Nagelkerke *R*^2^) of the variance in pregnancy rates and correctly classified 74% of cases. The sensitivity was 64.1%, specificity was 80.3%, positive predictive value was 67.6%, and negative predictive value was 77.8%. Of the predictor variables, only two were statistically significant: intraspecific insemination and Jenny (Table 2). Intraspecific insemination showed 0.5 times lower odds of resulting in a pregnancy than interspecific insemination, while jennies had 0.25 times lower odds of becoming pregnant than mares.

### 3.2. Day and Diameter at First Embryo Detection

The mean day of first embryo detection was 10.9 ± 1.1 (n = 16), 10.7 ± 0.8 (n = 7), 10.7 ± 0.7 (n = 13), and 10.9 ± 0.9 (n = 7) for the horse, mule, donkey, and hinny embryos, respectively (*p* < 0.05).

The mean embryo diameter (mm, n = 43) at pregnancy diagnosis was 3.88 ± 1.59 (n = 23) and 3.74 ± 1.33 (n = 20) for mares and jennies, respectively, and 4.10 ± 1.43 (n = 23) and 3.49 ± 1.46 (n = 20) for stallion and jacks, respectively (*p* > 0.05). Table 3 shows the different embryo diameters recorded at the first day of embryo detection in horse and donkey females after insemination with horse and donkey semen, respectively.

Table 4 shows the days at the first embryo detection in horse and donkey females after insemination with horse and donkey semen, respectively.

### 3.3. Embryo Quality

The 42 intact recovered embryos were classified morphologically as Grade 1 or 2 [28] without differences according to genotype.

Figure 2 shows pictures of each embryo genotype.

## 4. Discussion

The mule is the only equine hybrid that has been truly domesticated by humans. Mules were largely used in agriculture and the military and maintain a role today as leisure and working animals in special conditions [2,3,4], whereas hinnies have always been much less common and diffused [6,7]. Probably because of the low economic interest in these two hybrids, studies on the outcome of mating or artificial insemination between donkeys and horses are very rare. In fact, this is the first study to directly compare pregnancy rates, the first day of embryo appearance and diameter, and in vitro embryo morphological quality after intraspecific and interspecific horse and donkey artificial insemination.

Artificial insemination is universally accepted as the most powerful method to improve animal breeding, genetic selection [29], and limit the spread of venereal diseases [30,31]. Recently, interest in donkey breeding, welfare, and nutrition has grown due to interest in donkey milk [32,33], and artificial insemination can be used to manage relatively large herds of animals [19,20,21] and preserve biodiversity [34].

The results obtained in this study, in the case of intraspecific AI with fresh extended semen, were not surprising: the pregnancy rate was significantly higher in horses (14/16, 87.5%) than in donkeys (11/21, 52.4%). The results obtained in both species were in accordance with the literature, where the average pregnancy rates following artificial insemination with fresh semen in horses ranged from 76% to 83% [35]. On the contrary, it is universally accepted that insemination is more complicated in donkeys, where lower pregnancy rates have been reported by several authors [17,36,37,38]. The jenny cervix has a different anatomical conformation from that of mares. In donkeys, the lumen of the cervix is narrowed and tortuous, plus the vaginal portion of the cervix may have various conformations that resemble the letters “L”, “C”, or “V” [5]. These aspects represent a challenge for routine intrauterine procedures such as AI. Moreover, jennies have a more pronounced physiologic, post-breeding uterine inflammatory reaction compared to mares, which is exacerbated when frozen semen is used [5,38,39]. In one of our previous studies, the pregnancy rates ranged between 47% and 56% when using fresh semen in different protocols for timed artificial insemination in a total of 90 milk-producing jennies [21]. Similarly, a pregnancy rate of 31% was reported when jennies underwent repeated AI using 1 × 10^9^ fresh spermatozoa [19]. Pregnancy rates of 73% and 40% were reported when inseminating a low number of jennies (15 for each group) with 1 × 10^9^ or 500 × 10^6^ fresh spermatozoa, respectively [16].

In this study, a lower pregnancy rate for jennies was also confirmed by pooling the results of the intra- and interspecific AI: globally, 20/30 (66.7%) and 19/70 (27.1%) of the mares and jennies were pregnant, respectively (*p* < 0.05). Interestingly, the same phenomenon was not observed in males, as 22/65 (33.8%) and 17/35 (48.6%) cycles in which artificial insemination was carried out with horse or donkey semen, respectively, resulted in pregnancies (*p* > 0.05). The first partial conclusion is that the present study confirmed the lower pregnancy rate achievable in jennies versus mares by artificial insemination, even when using fresh semen, and that this was not due to the donkey stallion fertility.

In the present study, the pregnancy rate when inseminating mares with fresh donkey semen (6/14, 42.9%) was similar to that in the donkey × donkey combination but lower than the findings currently reported in the literature. Indeed, Paolucci et al. [22] observed pregnancy rates from 62.5% (5/8) to 71.4% (5/7) when inseminating mares of different age classes with donkey semen. De Oliveira et al. [16] reported a pregnancy rate of 93% following artificial insemination of mares with fresh donkey semen at a dose of 500 × 10^6^ viable spermatozoa. Other results ranged from 52.2% up to 89.6% [23,40,41] when AI was performed on a large number of mares. The low number of mares employed in the present study likely explains the discrepancy with the literature. Hinny embryos (horse stallion × jenny) were the hardest to obtain: only 8 jennies over 49 cycles became pregnant, with a pregnancy rate of 16.3%. This is very similar to the 14.5% reported by Allen et al. [24], who submitted jennies to horse stallions through natural mating or AI over 159 estrous cycles. To the best of our knowledge, Allen et al.’s study [24] and our studies are the only ones investigating the outcome of interspecific artificial insemination or mating of jennies with horse stallions.

Taken together, the results of the different combinations of AI demonstrated that the pregnancy rate was significantly higher for intraspecific (25/37, 67.6%) than interspecific AI (14/63, 22.2%). Although data on this type of comparison are not available in the literature, pregnancy rates are likely to be higher when breeding occurs between individuals of the same species than between individuals with a different number of chromosomes. However, it is possible that this difference was influenced by the large percentage of horse stallion × jenny breeding evaluated in this study which, as already reported, resulted in the lowest pregnancy rate. Endometritis is a physiologic inflammation that occurs after mating in the horse; nevertheless, persistent post-breeding endometritis compromises the uterine environment and establishment of pregnancy [39]. Post-breeding endometritis is more commonly found in post-frozen semen AI and especially in jennies [38,39,42,43]. In this study, AI was performed with fresh semen and no differences in uterine inflammatory reactions after AI were observed, neither according to the male/female genotype nor the different AI combinations. For this reason, it seems unlikely to attribute the lower pregnancy rate observed in interspecific breeding to more pronounced post-breeding uterine inflammation. Although it is a characteristic of equids to be able to interbreed and give birth to offspring, even if they are sterile, it is possible that reproductive efficiency is reduced during interspecific breeding due to the different number of chromosomes in donkeys and horses. According to what has been reported in the literature, pregnancies were only obtained when the female of the partnership between domestic horses and wild asses or zebras was the horse [8], while no pregnancies were reported when mating a horse stallion with a wild female donkey or a zebra [8]. This tendency was confirmed in the present study and in the study by Allen et al. [24] in which the lowest pregnancy rate was obtained by inseminating jennies with horse stallion semen. In this case, it is possible that the lowest result obtained could be due to both the well-known reduced efficiency of AI in donkeys and the more difficult interaction between horse semen and donkey oocyte.

In this study, no differences were observed from the ultrasound scan comparison of the day and diameter of the first appearance of the embryos of the different genotypes.

These parameters were consistent with the literature for horse embryos [44,45,46,47,48] but slightly different from the data of the only study reporting the average diameter and day of first mule embryo detection: 4.6 ± 1.1 mm and day 9.5 ± 1.5 [22] versus 3.69 ± 1.77 mm and day 10.7 ± 0.8 in the present study, respectively. These differences were likely due to the power of the US machine employed and the timing of pregnancy detection in the two studies.

As already stated, this is the first manuscript to report the day and size of the hinny embryo at first US detection and directly compare the in vitro morphological quality of horse, donkey, mule, and hinny embryos. The diameter and day of detection, as well as the in vitro quality, did not differ among the four genotypes. Therefore, this study confirmed that hybrids, mostly hinnies, are more difficult to produce than horses and donkeys; however, the study findings also indicated that the quality of the young hybrid embryos was as good as the quality of the corresponding pure genotype embryos.

One of the limitations of this study was the discrepancy between the number of inseminations of jennies with horse stallion semen compared to the numbers of the other three combinations. This was due to the very low efficiency of horse stallion × jenny AI, which required more attempts to obtain enough hinny embryos to be statistically compared with the other three genotypes. A second limitation was that embryo quality was only assessed considering the morphology. Further comparative analyses will be performed on the frozen embryos.

## 5. Conclusions

This is the first study to directly compare pregnancy rates, the first day of embryo appearance and diameter, and in vitro embryo morphological quality after intraspecific and interspecific horse and donkey artificial insemination.

The results of the study confirmed the difficulty in obtaining pregnancies in donkeys compared to those commonly reached in horses and showed that intraspecific AI led to significatively higher pregnancy rates than interspecific AI. This difference seems not to be related to the post-breeding uterine reaction, which was similar in the different AI combinations. Nevertheless, the embryo developing rate and morphological quality at days 10–16 after ovulation were not different between the groups and between pure or hybrid embryos.

Further studies on the genomic and proteomic assets of the different pure and hybrid equid genotypes may contribute to explaining the genesis of such different outcomes of intra- and interspecific artificial insemination in domestic equids.

## Figures and Tables

**Table 1:** Pregnancy rates according to the genotype of the conceptus in horse and donkey females inseminated with horse and donkey semen.

Combinations	Pregnancy Rate (%)
Horse ♂ × Horse ♀ (Horse)	14/16 (87.5) ^a^
Horse ♂ × Donkey ♀ (Hinny)	8/49 (16.3) ^b^
Donkey ♂ × Horse ♀ (Mule)	6/14 (42.9) ^b,c^
Donkey ♂ × Donkey ♀ (Donkey)	11/21 (52.4) ^c^

^a,b,c:^ Different superscripts in the same column indicate a significant difference at *p* < 0.05.

**Table 2:** Logistic regression predicting the likelihood of pregnancy based on intraspecific or interspecific insemination or species of the inseminated female (jenny or mare).

95% CI for Odds Ratio					
	B (SE)	Lower	Odds Ratio	Upper	*p*
Constant	1.694 (0.55)				
Intraspecific insemination	−1.98 (0.55)	0.06	0.15	0.40	0.0001
Jenny	−1.60 (0.55)	0.07	0.25	0.59	0.004

**Table 3:** Embryo diameters (mean ± SD) in mm at pregnancy diagnosis in horse and donkey females inseminated with horse and donkey semen, according to the day of embryo detection. (*p* > 0.05).

Embryo Æ (Mean ± SD)					
Combination	Day 10	Day 11	Day 12	Day 14	Total
Horse ♂ × Horse ♀	3.45 ± 1.02 (n = 8)	3.8 ± 0.32 (n = 4)	5.33 ± 3.21 (n = 3)	4.7 ± 0 (n = 1)	3.97 ± 1.56 (n = 16)
Horse ♂ × Donkey ♀	3.93 ± 1.10 (n = 3)	4.83 ± 1.44 (n = 3)	4.5 ± 0 (n = 1)		4.40 ± 1.14 (n = 7)
Donkey ♂ × Donkey ♀	2.87 ± 1.06 (n = 6)	3.72 ± 1.07 (n = 5)	4.10 ± 0.99 (n = 2)		3.38 ± 1.33 (n = 13)
Donkey ♂ × Horse ♀	3.30 ± 0.44 (n = 3)	3.75 ± 4.03 (n = 2)	4.2 ± 1.13 (n = 2)		3.69 ± 1.77 (n = 7)
Total	3.32 ± 0.96 (n = 20)	3.99 ± 1.64 (n = 14)	4.63 ± 1.9 (n = 8)	4.7 ± 0 (n = 1)	3.82 ± 1.46 (n = 43)

**Table 4:** Day of first embryo detection (mean ± SD) in horse and donkey females inseminated with horse and donkey semen (*p* > 0.05).

Combination	Day of First Pregnancy Diagnosis
Horse ♂ × Horse ♀	10.88 ± 1.15 (n = 16)
Horse ♂ × Donkey ♀	10.71 ± 0.76 (n = 7)
Donkey ♂ × Donkey ♀	10.69 ± 0.75 (n = 13)
Donkey ♂ × Horse ♀	10.86 ± 0.90 (n = 7)
Total	10.79 ± 0.91 (n = 43)

## Data Availability

Data available on reasonable request.
